# Gibbsian Thermodynamic Study of Capillary Meniscus Depth

**DOI:** 10.1038/s41598-018-36514-w

**Published:** 2019-01-24

**Authors:** Fatemeh Eslami, Janet A. W. Elliott

**Affiliations:** 1 Department of Process Engineering, Faculty of Chemical Engineering, Tarbiat Modares University, Tehran, Iran; 2 Department of Chemical and Materials Engineering, University of Alberta, Edmonton AB, T6G 1H9 Canada

## Abstract

In the presence of gravity or other external fields, liquid surface curvature deviates from a spherical shape and the surface configuration can be found by numerical integration of the Young–Laplace equation and the typical initial point for integration is the apex of the interface. The meniscus shape in large Bond number systems, which have the central portion of the interface flattened, cannot be determined with the apex as the initial point for integration. Here we find the depth of capillary menisci by considering an initial point for integration to be at the three-phase-contact-line (*TPCL*) and evaluate the curvature at the *TPCL* by free energy analysis and inspect the effect of different parameters on the interface shape. A new parameter—which is the deviation of equilibrium curvature at the *TPCL* from the spherical shape (SR)—is introduced and inspected and it was found that at a Bond number of 13 the maximum deviation, approximately 0.8 of spherical curvature, takes place while for large enough Bond numbers the curvature at the three-phase contact line is near the spherical shape (0.95 < SR < 1). A potential application of this approach is to measure the capillary rise at the *TPCL* to find the surface tension in high Bond number systems such as those with low surface/interfacial tensions.

## Introduction

Finding the equilibrium shape of fluid interfaces has been frequently studied due to its importance in surface science^1–17^. The fluid surface tension and the contact angle that the fluid makes with a solid are two important surface parameters that directly relate to the fluid interface shapes. Sessile drops, pendent drops, bubbles, foams and capillary menisci are some of the important fluid interfaces that we are involved with ubiquitously. The Young–Laplace equation is the most famous relation that governs the equilibrium shape of fluid interfaces. It connects a capillary pressure (the inner and outer phase pressure difference across the curved interface) to the interfacial tension between the two fluid phases and the geometric property of the interface—its mean curvature. When two fluid phases meet a solid phase, the competition between their surface energies leads to the formation of a capillary meniscus.

The calculation of equilibrium shapes of capillary menisci has been done for two main reasons: (*a*) determination of surface/interfacial tensions and contact angles, and (*b*) determination of capillary forces.

The first calculation of fluid interface shape is attributed to Bashforth and Adams^18^. They performed numerical integration of the Young–Laplace equation for sessile drops. They non-dimensionalized the Young–Laplace equation with *b—*the mean curvature of the surface at the apex—and used the apex as the starting point for numerical integration and provided the results of their calculation in several tables for different values of $\frac{{\rm{\Delta }}\rho g{b}^{2}}{{\gamma }^{LV}}$ where *g*, Δ*ρ* and *γ*^*LV*^ are gravitational acceleration, liquid–vapor density difference, and liquid–vapor surface tension, respectively. Later, Padday and Pitt^14^ completed their work by changing the starting point to be at turning angle *φ* = 270° and non-dimensionalized the equation with surface curvature at this starting point. Padday and Pitt mentioned that for menisci that never intersect the axis of symmetry, such as liquid bridges between two solids, the mean curvature at the apex has no real significance and that this difficulty can be overcome by moving the integration starting point. Bucher^1^ published an excellent review for interface configurations and classified these systems into sessile and pendent drops, emergent and captive bubbles, fluid bridges and holms (the bridging fluid between a solid and a bulk fluid). He summarized the integration starting point for these interfaces, and for sessile and pendent drops and emergent and captive bubbles the apex of the interface is chosen to be the initial point for integration. Rothenberg *et al*.^5^ minimized an objective function—the difference between the experimental and theoretical shapes of the sessile and pendent drops—and as a result of this optimization obtained the surface tensions and contact angles of fluid interfaces. Grzybowski *et al*.^10^ considered several floating objects of approximately millimeter size and different hydrophobicities at the interface of water and perfluorodecalin. They calculated the interface shape of menisci by two methods: analytic solution of the Young–Laplace equation and use of the finite element method in the case of no analytical solution with the former method. They found the energy profile and capillary forces of the system from the calculated interface shape.

Asekomhe and Elliott^19^ computed the shapes of fluid menisci used to infer line tensions from capillary rise in a conical tube and showed that considering the effect of gravity on the liquid–vapor interface shape changed the inferred solid–liquid–vapor line tension values by up to 50%. Danov *et al*.^2^ investigated the capillary meniscus around an electrically charged particle deforming a fluid interface and found that the meniscus deformation for small enough particles is the sum of deformations due to gravity and electric forces individually. Kuchin *et al*.^11^ carefully examined the shape of the capillary meniscus in the transition zone—where the meniscus meets a thin liquid film at the wall so that both the disjoining/conjoining pressure and the capillary pressure play important roles in deforming the meniscus shape. They mentioned that disjoining/conjoining pressure is the dominant surface force in the case of thin liquid films and capillary pressure is the prevailing force in the spherical part of the meniscus. Rayleigh^20^ investigated the capillary rise method in order to measure surface tension and proposed two approximate relations: one for systems with Bond numbers smaller than 0.04 and one for systems with Bond numbers larger than 0.04.

Chatterjee^21^ investigated two coupled menisci in a capillary and by fitting the upper meniscus curvature to the experimental data predicted the shape of the bottom meniscus. Ratcliffe *et al*.^17^ explored the shape of a drop trapped in a constriction. They solved the Young–Laplace equation for the fluid–fluid interface with the constriction imposing its shape on the solid–liquid interface. Lubarda and Talke^12^ assumed ellipsoidal shapes for sessile drops in the presence of gravity and did the minimization of free energy to get the equilibrium height and droplet spreading. They compared their ellipsoidal results with the numerical solution and found that the ellipsoidal shape assumption is accurate for droplets with contact angles smaller than 120° and for droplets with sizes on the order of the capillary length.

Hartland and Hartley calculated the external meniscus shape of fluid which has a horizontal part far from the deforming body^22^. They considered the vertical axis of the deforming body and the horizontal axis along the horizontal part of the meniscus to be the z and x axes of their system. In order to be able to solve the problem they simplified the problem by considering *dz/dx* ≪1 and found the solution to be a Bessel function. They further did the integration by starting the integration from arbitrarily small *φ* and tabulated their results. In order to use their calculation approach a parameter must be fitted to experimental measurement of the system of interest.

Calculation of meniscus shape and specifically the shape of a liquid droplet is also of great interest in optical science^3,23,24^. Due to the symmetry of liquid droplets they act like lenses, and today they attract attention as adaptive lenses especially in miniaturized systems where surface forces are dominant. Electrowetting and dielectrophoretic operations are two manipulations that can change meniscus curvature and consequently the focal length of these lenses. Ren *et al*.^3^ did several experiments for small droplets and found that even for vertical lenses, the gravity will affect the symmetry of droplets negligibly. As a result, any two immiscible liquids regardless of their density mismatch can be used as an optical lens.

Contact angle measurement in biochemical systems—where only a small volume of matter is available—is of great importance. In order to prevent contact line pinning due to evaporation and also to avoid contamination of the biochemical material, instead of using the sessile drop method, microscopic imaging of the capillary meniscus in a retainer such as a test tube, is suggested^24^. However, in case of a cylindrical retainer there are some difficulties due to image distortion. Cheong *et al*. showed that knowing the shape of the meniscus (radius and height) is enough to measure the contact angle. However, they assumed a spherical shape for the meniscus and verified their calculated contact angles with measured ones.

Generally speaking, meniscus shape calculation has been done by various methods and for numerous applications. In small capillaries (small Bond numbers) a capillary meniscus forms part of a sphere and the depth of the meniscus scales with the capillary radius. As the Bond number increases, part of the meniscus becomes flattened due to gravitational effects. In order to find the capillary depth in menisci that are flattened in the center, the initial point for numerical integration cannot be the meniscus apex. As a result, we choose the initial point to be at the three-phase contact line and we inspect the curvature at this point by introducing a parameter which shows the deviation of equilibrium curvature at the three-phase contact line from the spherical shape (*SR*).

## Theory

**Figure 1:**
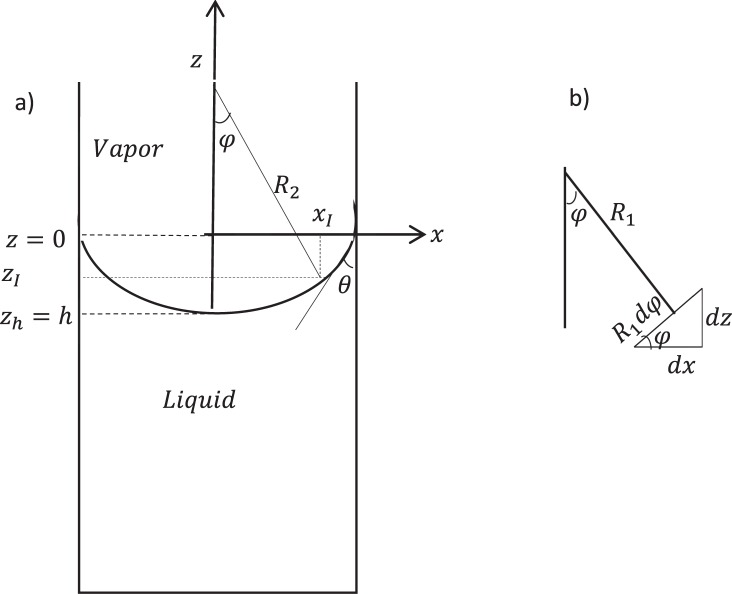
(**a**) Capillary meniscus shape; (**b**) meridional radius of curvature *R*_1_ and related geometry.

The system of interest is shown in Fig. 1 which is a capillary partially filled with a single component *i* as a liquid phase which coexists with its vapor phase. The system is symmetric so the axis of symmetry is the *z* axis and the three-phase contact line in the two-dimensional system is the vertical location of the *x* axis. We are going to find the meniscus depth of fluid (*z*_*h*_ = *h*). At equilibrium the system has constant temperature (*T*^*L*^ = *T*^*V*^) and the Young–Laplace equation governs the pressure difference across the fluid interface:

1 $${P}^{V}{|}_{{z}_{I}}-{P}^{L}{|}_{{z}_{I}}={\gamma }^{LV}(\frac{1}{{R}_{1}}+\frac{1}{{R}_{2}})=\frac{2{\gamma }^{LV}}{{R}_{m}}$$

where *P*^*V*^ and *P*^*L*^ are the vapor and liquid pressures, *z* is the elevation with respect to the three-phase contact line, *R*_1_ and *R*_2_ are the meridional and azimuthal principal radii of curvature of the interface with an axis of rotational symmetry, *R*_*m*_ is the mean radius of surface curvature and subscript *I* indicates that a property is referred to the interface. The following equation is the third equilibrium condition which relates the chemical potential to the elevation:

2 $${\mu }_{i}(T,P{|}_{z})={\mu }_{i}(T,P{|}_{{z}_{ref}})-{M}_{i}\,g(z-{z}_{ref})$$

where *μ*_*i*_ is the chemical potential of component *i*, *M*_*i*_ is the molar mass of component *i* and subscript *ref* indicates the reference point. In order to use these equilibrium conditions for our system we have to use the equations of state. If we assume the liquid phase to be an incompressible liquid and the gas phase to be an ideal gas, then we will have:

3 $${\mu }_{i}^{L}(T,{P}^{L}{|}_{z})={\mu }_{i}^{L}(T,{P}^{L}{|}_{{z}_{ref}})+{v}_{\infty }^{L}({P}^{L}{|}_{z}-{P}^{L}{|}_{{z}_{ref}})$$

4 $${\mu }_{i}^{V}(T,{P}^{V}{|}_{z})={\mu }_{i}^{V}(T,{P}^{V}{|}_{{z}_{ref}})+{R}_{u}T{\mathtt{l}}{\mathtt{n}}(\frac{{{P}}^{{V}}{|}_{{z}}}{{{P}}^{{V}}{|}_{{{z}}_{{r}{e}{f}}}})$$

where ${v}_{\infty }^{L\,}$ is the liquid molar volume at the saturation condition and *R*_*u*_ is the universal gas constant.

Substituting Equation (2) in (3) results in:

5 $${P}^{L}{|}_{z}={P}^{L}{|}_{{z}_{ref}}-{\rho }^{L}g(z-{z}_{ref})$$

where ${\rho }^{L}$ is the liquid phase density. And substituting Equation (2) in (4) leads to:

6 $${P}^{V}{|}_{z}={P}^{V}{|}_{{z}_{ref}}{\mathtt{e}}{\mathtt{x}}{\mathtt{p}}(\frac{{-}{M}{g}({z}{-}{{z}}_{{r}{e}{f}})}{{{R}}_{{u}}{T}})$$

Since we are dealing with small (*z *− *z*_*ref*_) and at small *x*, exp(*x*) ≈ 1 + x, we can simplify Equation (6) to:

7 $${P}^{V}{|}_{z}={P}^{V}{|}_{{z}_{ref}}-{\rho }^{V}g(z-{z}_{ref})$$

where *ρ*^*V*^ is the vapor phase density. By subtracting Equation (5) from (7) we will have:

8 $$({P}^{V}-{P}^{L}){|}_{z}=({P}^{V}-{P}^{L}){|}_{{z}_{ref}}+{\rm{\Delta }}\rho g(z-{z}_{ref})$$

where ${\rm{\Delta }}\rho ={\rho }^{L}-{\rho }^{V}$. If we combine Equation (8) with Equation (1) then it can be used for the liquid–vapor interface and the meniscus shape can be calculated from

9 $${\gamma }^{LV}(\frac{1}{{R}_{1}}+\frac{1}{{R}_{2}})=\frac{2{\gamma }^{LV}}{{R}_{m0}}+{\rm{\Delta }}\rho g(z-{z}_{ref})$$

where *R*_*m*0_ is the mean radius of curvature at the reference point. Following the technique initiated by Bashforth^19^ and Adams and used later by Ward and Sasges^8^, the following relations relate the meridional and azimuthal principal radii of curvature:

10 $${\mathtt{s}}{\mathtt{i}}{\mathtt{n}}\,\phi {\mathtt{=}}\frac{{{x}}_{{I}}}{{{R}}_{{2}}}$$

11 $$\sin \,\phi {\mathtt{=}}\frac{{d}{{z}}_{{I}}}{{{R}}_{{1}}{(}\phi {)}{d}\phi }$$

12 $$\cos \,\phi {\mathtt{=}}\frac{{d}{{x}}_{{I}}}{{{R}}_{{1}}{(}\phi {)}{d}\phi }$$

where *φ* is the turning angle. Substituting Equations (10–12) in Equation (9), the following differential equations govern the interfacial shape:

13 $$d{x}_{I}=\frac{{\mathtt{c}}{\mathtt{o}}{\mathtt{s}}\phi \,{d}\phi }{\frac{2}{{R}_{m0}}+\frac{{\rm{\Delta }}\rho g({z}_{I}-{z}_{ref})}{{\gamma }^{LV}}-\frac{{\mathtt{s}}{\mathtt{i}}{\mathtt{n}}\phi }{{x}_{I}}}$$

14 $$d{z}_{I}=\frac{{\mathtt{s}}{\mathtt{i}}{\mathtt{n}}\phi \,{d}\phi }{\frac{2}{{R}_{m0}}+\frac{{\rm{\Delta }}\rho g({z}_{I}-{z}_{ref})}{{\gamma }^{LV}}-\frac{{\mathtt{s}}{\mathtt{i}}{\mathtt{n}}\phi }{{x}_{I}}}$$

where *R*_*m0*_ is the curvature of the meniscus at the three-phase contact line:

15 $${R}_{m0}=\frac{2{\gamma }^{LV}}{{P}^{V}{|}_{z=0}-{P}^{L}{|}_{z=0}}$$

We assume the reference point to be at the three-phase contact line (*z*_*ref*_ = 0) and that ${P}^{V}{|}_{z=h}={P}^{\infty }$, the saturation pressure, and then by making use of Equation (7) ${P}^{V}{|}_{z=0}={P}^{\infty }+{\rho }^{V}gh$. In addition, by non-dimensionalizing the terms *x*, *z*, *R*_*m0*_ with respect to the capillary radius *i*.*e*. $x^{\prime} =\frac{x}{r}$ Equations (13) and (14) will simplify to:

16 $$d{x}_{I}^{{\rm{^{\prime} }}}=\frac{{\mathtt{c}}{\mathtt{o}}{\mathtt{s}}\phi \,{d}\phi }{\frac{2}{{R}_{m0}^{{\rm{^{\prime} }}}}+B{z}_{I}^{{\rm{^{\prime} }}}-\frac{{\mathtt{s}}{\mathtt{i}}{\mathtt{n}}\phi }{{{x}^{{\rm{^{\prime} }}}}_{I}}}$$

17 $$d{z}_{I}^{{\rm{^{\prime} }}}=\frac{{\mathtt{s}}{\mathtt{i}}{\mathtt{n}}\phi \,{d}\phi }{\frac{2}{{{R}^{{\rm{^{\prime} }}}}_{m{\rm{o}}}}+B{z}_{I}^{{\rm{^{\prime} }}}-\frac{{\mathtt{s}}{\mathtt{i}}{\mathtt{n}}\phi }{{{x}^{{\rm{^{\prime} }}}}_{I}}}$$

where *B* is the Bond number. When in a system both the capillary force and the gravitational force are appreciable, there is a competition between them. This competition can be evaluated by a non-dimensional number—the Bond number—and it is defined as follows:

18 $$B=\frac{{\rm{\Delta }}\rho g{L}^{2}}{{\gamma }^{LV}}$$

where *L* is a characteristic length scale of the system which in capillaries is usually assumed to be the capillary radius. Most of the previous calculations^5,7,8,12,16,19,21,24^ use the curvature at the meniscus apex as an iterated boundary condition to solve the differential equations. However, this approach cannot be used to predict the shape of a meniscus with a flat portion at its center which is the case for systems with large Bond numbers. The challenge in numerically solving this problem upon moving the initial point for integration is to devise an integration procedure using an iterated  boundary condition and stopping conditions that yields a unique solution.

Now, in order to fully overcome this shortcoming, we consider the starting point for integration to be at the three-phase contact line (*z* = 0). As a result, the equilibrium meniscus shape will be found by numerically integrating the differential Equations (16) and (17) from $\phi =\frac{\pi }{2}-\theta$ to $\phi =0\,$ with the starting point of ${x}_{I}^{{\rm{^{\prime} }}}=1$ and ${z}_{I}^{{\rm{^{\prime} }}}=0$. However, in order to do the integration we still have one more unknown which is the curvature at the three-phase contact line, ${R^{\prime} }_{m{\rm{o}}}$.

We try to find it by assuming an initial value for ${R^{\prime} }_{m{\rm{o}}}$ and doing the integration. If the correct shape is found then $\frac{d{z^{\prime} }_{I}}{d\phi }{|}_{\phi =0}\,$ should be equal to zero in a smooth way. However, this approach will not lead to a unique $\,{R^{\prime} }_{m{\rm{o}}}$ hence it is not adequate. Here we found an interval for ${R^{\prime} }_{m{\rm{o}}}$ which satisfies all the equilibrium conditions while in order to find a unique mathematical and physical solution, the solution should both satisfy all the equilibrium conditions and obtain the lowest free energy subject to the system constraints. In order to choose an appropriate ${R^{\prime} }_{m{\rm{o}}}$ from this interval we use the free energy analysis^25–29^. It is worth mentioning that considering curvature length (*s*) as an integrating variable a similar result will be obtained. In this case the following relations exist between the *x*, *z*, *φ* and *s*:

19 $$\begin{array}{cc}\frac{d{x}_{I}^{{\rm{^{\prime} }}}}{d{s}^{{\rm{^{\prime} }}}}=\,\cos \,\phi  & \frac{d{z}_{I}^{{\rm{^{\prime} }}}}{d{s}^{{\rm{^{\prime} }}}}=\,\sin \,\phi \end{array}$$

20 $$\frac{d\phi }{d{s}^{{\rm{^{\prime} }}}}=\frac{2}{{{R}^{{\rm{^{\prime} }}}}_{m{\rm{o}}}}-\frac{{\mathtt{s}}{\mathtt{i}}{\mathtt{n}}\phi }{{x}_{I}^{{\rm{^{\prime} }}}}+B{z}_{I}^{{\rm{^{\prime} }}}$$

This set of differential equations should be solved from ${s^{\prime} }_{c}$ (curvature length at the three phase contact line) to $\,s^{\prime} =0$ with the starting point of ${x}_{I}^{{\rm{^{\prime} }}}=1$ and ${z}_{I}^{{\rm{^{\prime} }}}=0$ while ${s}_{\,c\,}^{{\rm{^{\prime} }}}$ is not known in advance. Here, a value is guessed for ${s^{\prime} }_{c}$ and ${R^{\prime} }_{m0}$ and the correct meniscus profile will be obtained if *x*′ = 0 at *φ* = 0.

### Free energy calculation method

In order to choose an appropriate ${R^{\prime} }_{m0}$ from the acceptable interval (for which all conditions for equilibrium are satisfied) we analyze the free energy of the system to find which ${R^{\prime} }_{m0}$ results in the lowest system free energy among them and hence is the stable ${R^{\prime} }_{m0}$.

**Figure 2:**
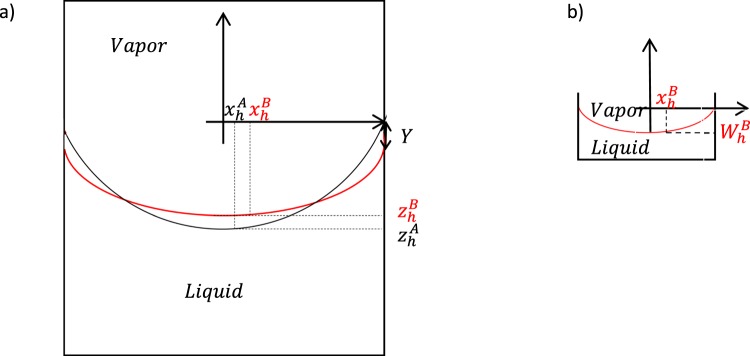
(**a**) Two different menisci for a fixed capillary size and contact angle with two different values for ${R}_{m0}$ in the coordinates of meniscus *A* as a reference. (**b**) Meniscus *B* in its coordinate system.

Figure 2 shows the system of interest which consists of liquid and vapor phases and *LV*, *SL* and *SV* interfaces. There are two meniscus profiles in this figure which we will name states *A* and *B*. We are going to analyze the free energy difference of *F*^*B*^ − *F*^*A*^ near equilibrium where both cases of *A* and *B* have the same volume of water with two different values of ${R^{\prime} }_{m0}$. By changing the value of ${R^{\prime} }_{m0}$ the corresponding ${F}^{B}$ is changed and ${F}^{A}$ acts as a reference free energy. *x*_*h*_ and *z*_*h*_ are the values of *x* and *z* at the point closest to the capillary wall at which the turning angle *φ* = 0. In the case of a meniscus without a flat portion this point would be at the capillary center, but for a meniscus with a flat portion this point is not at the center but rather at the furthest extent of the flat portion. The reference case *A* is chosen in a way that it has the lowest *z*_*h*_. Since both states *A* and *B* have the same volume of water with two different ${R^{\prime} }_{m0}$, *Y* is the displacement of the origin of the coordinate system of meniscus *B* with respect to the origin of the coordinate system of meniscus *A* due to the liquid volume being the same for *A* and *B:*

21 $${V}^{L}=\pi {\int }_{{z}_{h}^{A}}^{0}({r}^{2}-{{x}_{h}^{A}}^{2})dz={\int }_{{z}_{h}^{B}}^{Y}\pi ({r}^{2}-{{x}_{h}^{B}}^{2})dz+\pi {r}^{2}({z}_{h}^{B}-{z}_{h}^{A})$$

In Fig. 2(b) the meniscus *B* is shown in its coordinate system where $\,{W}_{h}^{B}$ is analogous to ${z}_{h}^{B}$ but in the coordinates of meniscus *B*. By non-dimensionalizing of length scales and by knowing that ${W}_{h}^{B}+Y={z}_{h}^{B}$, the non-dimensionalized *Y*′ would be equal to:

22 $${Y}^{{\rm{^{\prime} }}}={\int }_{{{z}^{{\rm{^{\prime} }}}}_{h}^{A}}^{0}(1-{{{x}^{{\rm{^{\prime} }}}}_{h}^{A}}^{2})d{z}^{{\rm{^{\prime} }}}-{\int }_{{{W}^{{\rm{^{\prime} }}}}_{h}^{B}}^{0}(1-{{{x}^{{\rm{^{\prime} }}}}_{h}^{B}}^{2})d{z}^{{\rm{^{\prime} }}}+{{z}^{{\rm{^{\prime} }}}}_{h}^{A}-{{W}^{{\rm{^{\prime} }}}}_{h}^{B}$$

The system has constant volume therefore Helmholtz free energy (*F*) plus potential energy (*E*)^30^ governs the system:

23 $$F+E={F}^{L}+{F}^{V}+{F}^{LV}+{F}^{SL}+{F}^{SV}+{E}^{L}+{E}^{V}$$

24 $$\begin{array}{ccc}{F}^{B} & = & {\int }_{{z}_{h}^{A}}^{{z}_{h}^{B}}({f}^{L}+{e}^{L})\pi ({r}^{2})dz+{\int }_{{z}_{h}^{B}}^{Y}({f}^{L}+{e}^{L})\pi ({r}^{2}-{{x}_{h}^{B}}^{2})dz+\,{\int }_{{z}_{h}^{B}}^{Y}({f}^{V}+{e}^{V})\pi {{x}_{h}^{B}}^{2}dz\\  &  & +{\int }_{Y}^{0}({f}^{V}+{e}^{V})\pi {r}^{2}dz+{\int }_{{z}_{h}^{B}}^{Y}{f}^{LV}2\pi {x}_{h}^{B}dz+{f}^{LV}\pi {{x}_{h}^{B}}^{2}\\  &  & +{\int }_{{z}_{h}^{A}}^{Y}{f}^{SL}2\pi rdz+{\int }_{Y}^{0}{f}^{SV}2\pi rdz\end{array}$$

25 $${F}^{A}={\int }_{{z}_{h}^{A}}^{0}{f}^{L}\pi ({r}^{2}-{{x}_{h}^{A}}^{2})dz+{\int }_{{z}_{h}^{A}}^{0}{f}^{V}\pi {{x}_{h}^{A}}^{2}dz+{\int }_{{z}_{h}^{A}}^{0}{f}^{LV}2\pi {x}_{h}^{A}dz+{f}^{LV}\pi {{x}_{h}^{A}}^{2}+{\int }_{{z}_{h}^{A}}^{0}{f}^{SL}2\pi rdz$$

where for the bulk volume (liquid and vapor phase) the intensive Helmholtz energy (*f*) is given by the following and *n* is the number of moles per unit volume of bulk phases. Using Equations (3–7) we will have:

26 $$\begin{array}{ccc}{f}^{L}(z) & = & -{P}^{L}{|}_{z}+{n}^{L}{\mu }^{L}({P}^{L}{|}_{z})=-\,{P}^{L}{|}_{z=0}+{\rho }^{L}gz+{n}^{L}[{\mu }^{L}({P}^{L}{|}_{z=0})-{v}_{\infty }^{L}{\rho }^{L}gz]\\  & = & -{P}^{L}{|}_{z=0}+{n}^{L}{\mu }^{L}({P}^{L}{|}_{z=0})\end{array}$$

27 $$\begin{array}{ccc}{f}^{V}(z) & = & -{P}^{V}{|}_{z}+{n}^{V}{\mu }^{V}({P}^{V}{|}_{z})=-{P}^{V}{|}_{z=0}+{\rho }^{V}gz+{n}^{V}[{\mu }^{V}({P}^{V}{|}_{z=0})-Mgz]\\  & = & -{P}^{V}{|}_{z=0}+{n}^{V}{\mu }^{V}({P}^{V}{|}_{z=0})\end{array}$$

For the interfaces (*j* = *SL*, *SV* and *LV*) the intensive Helmholtz energy is given by:

28 $${f}^{j}(z)={\gamma }^{j}+{n}^{j}{\mu }_{i}^{j}$$

where *n* is the adsorbed number of moles per unit area and since we neglect any adsorption effects in the system, for all the three interfaces $\,{f}^{j}(z)\approx {\gamma }^{j}$. The intensive potential energy (*e*) for the bulk phases is:

29 $${e}^{j}(z)={n}^{j}Mgz$$

At equilibrium the Young equation governs the contact angle at the three-phase contact line:

30 $${\gamma }^{SV}-{\gamma }^{SL}={\gamma }^{LV}cos\theta $$

By having a fixed coordinate system of the reference case for all menisci and considering menisci displacement with respect to that fixed coordinate system, based on Equations (26) and (27) we will have for bulk phases *j* = *L*,*V*:

31 $${f}^{j,A}{|}_{z}=-\,{P}^{j,A}{|}_{z=0}+{n}^{j,A}{\mu }^{j,A}({P}^{j,A}{|}_{z=0})$$

32 $${f}^{j,B}{|}_{z}=-\,{P}^{j,{\boldsymbol{B}}}{|}_{z=Y}+{n}^{j,B}{\mu }^{j,B}({P}^{j,A}{|}_{z=Y})$$

For more clarity we separate the total energy difference into three parts:

33 $${F}^{B}-{F}^{A}={\rm{\Delta }}{F}^{surf}+{\rm{\Delta }}{F}^{bulk}+{\rm{\Delta }}{F}^{pot}$$

with a portion due to differences in interfacial free energies, Δ*F*^*surf*^ a portion due to differences in bulk liquid and vapor phase free energies, Δ*F*^*bulk*^, and a portion due to differences in potential energies, Δ*F*^*pot*^. The surface energy difference is the main part of the total energy difference and is based on the combination of interface energies. By using Equations (24), (25), (28) and (30) and non-dimensionalizing the length scales the surface free energy difference is found to be equal to:

34 $$\begin{array}{ccc}{\rm{\Delta }}{F}^{surf} & = & {\rm{\Delta }}{F}^{LV}+{\rm{\Delta }}{F}^{SL}+{\rm{\Delta }}{F}^{SV}\\  & = & \pi {\gamma }^{LV}{r}^{2}({\int }_{{{W}^{{\rm{^{\prime} }}}}_{h}^{B}}^{0}2{{x}^{{\rm{^{\prime} }}}}_{h}^{B}d{z}^{{\rm{^{\prime} }}}-{\int }_{{{z}^{{\rm{^{\prime} }}}}_{h}^{A}}^{0}2{{x}^{{\rm{^{\prime} }}}}_{h}^{A}d{z}^{{\rm{^{\prime} }}}+{\int }_{{Y}^{{\rm{^{\prime} }}}}^{0}2cos\theta d{z}^{{\rm{^{\prime} }}}+{{{x}^{{\rm{^{\prime} }}}}_{h}^{B}}^{2}-{{{x}^{{\rm{^{\prime} }}}}_{h}^{A}}^{2})\end{array}$$

The bulk energy difference relates to Helmholtz free energy of the bulk phases. Based on Equations (31) and (32) the *f* ^*j*^ do not depend on *z* so we can take them out of the integral. Since *V*^*L*^ and total volumes are constant for all the menisci we can simplify the bulk energy as follows:

35 $$\begin{array}{rcl}{\rm{\Delta }}{F}^{bulk} & = & {\rm{\Delta }}{F}^{L}+{\rm{\Delta }}{F}^{V}={V}^{L}(-{P}^{L,B}{|}_{z=Y}+{P}^{L,A}{|}_{z=0})+{V}^{V}(-{P}^{V,B}{|}_{z=Y}+{P}^{V,A}{|}_{z=0})\\  &  & +\,{V}^{L}{n}^{L}[{\mu }^{L,B}({P}^{L,B}{|}_{z=Y})-{\mu }^{L,A}({P}^{L,A}{|}_{z=0})]\\  &  & +\,{V}^{V}{n}^{V}[{\mu }^{V,B}({P}^{V,B}{|}_{z=Y})-{\mu }^{V,A}({P}^{V,A}{|}_{z=0})]\end{array}$$

By making use of Equations (3) and (4), the bulk free energy difference term will be equal to zero:

36 $$\begin{array}{rcl}{\rm{\Delta }}{F}^{bulk} & = & {\rm{\Delta }}{F}^{L}+{\rm{\Delta }}{F}^{V}={V}^{L}(-{P}^{L,B}{|}_{z=Y}+{P}^{L,A}{|}_{z=0})+{V}^{V}(-{P}^{V,B}{|}_{z=Y}+{P}^{V,A}{|}_{z=0})\\  &  & +\,{V}^{L}{n}^{L}({v}^{\infty }({P}^{L,B}{|}_{z=Y}-{P}^{L,A}{|}_{z=0}))+{V}^{V}{n}^{V}(-MgY)=0\end{array}$$

The potential energy difference is obtained by:

37 $$\begin{array}{ccc}{\rm{\Delta }}{F}^{pot} & = & \pi {r}^{4}({\int }_{{{z}^{{\rm{^{\prime} }}}}_{h}^{A}}^{{{z}^{{\rm{^{\prime} }}}}_{h}^{B}}{\rho }^{L}g{z}^{{\rm{^{\prime} }}}d{z}^{{\rm{^{\prime} }}}+{\int }_{{{z}^{{\rm{^{\prime} }}}}_{h}^{B}}^{Y}{\rho }^{L}g{z}^{{\rm{^{\prime} }}}(1-{{{x}^{{\rm{^{\prime} }}}}_{h}^{B}}^{2})d{z}^{{\rm{^{\prime} }}}+{\int }_{{z{\rm{^{\prime} }}}_{h}^{B}}^{Y}{\rho }^{V}g{z}^{{\rm{^{\prime} }}}{{{x}^{{\rm{^{\prime} }}}}_{h}^{B}}^{2}d{z}^{{\rm{^{\prime} }}}\\  &  & +{\int }_{{Y}^{{\rm{^{\prime} }}}}^{0}{\rho }^{V}g{z}^{{\rm{^{\prime} }}}d{z}^{{\rm{^{\prime} }}}-{\int }_{{{z}^{{\rm{^{\prime} }}}}_{h}^{A}}^{0}{\rho }^{L}g{z}^{{\rm{^{\prime} }}}(1-{{{x}^{{\rm{^{\prime} }}}}_{h}^{A}}^{2})d{z}^{{\rm{^{\prime} }}}-{\int }_{z{{\rm{^{\prime} }}}_{h}^{A}}^{0}{\rho }^{V}g{z}^{{\rm{^{\prime} }}}{{{x}^{{\rm{^{\prime} }}}}_{h}^{A}}^{2}d{z}^{{\rm{^{\prime} }}})\end{array}$$

By calculating the total free energy and plotting it for different values of $\,{R^{\prime} }_{m0}$, the most stable fluid curvature at the three-phase contact line will be evaluated.

## Results and Discussion

### Liquid water – water vapor system

Here we found the meniscus shape for liquid water in a capillary with capillary radius *r* and a contact angle of *θ*. The physical properties of the system corresponding to *T* = 25 °C are^31^: P^∞^ = 3170 Pa, ${\rho }^{L}=997.04\,\frac{{\rm{kg}}}{{{\rm{m}}}^{3}}$, ${\rho }^{V}=0.023\frac{{\rm{kg}}}{{{\rm{m}}}^{3}}$, ${\gamma }^{LV}=0.0727\frac{{\rm{J}}}{{{\rm{m}}}^{2}}$. As was mentioned in the previous section, in order to find the meniscus shape and depth even for capillaries with large Bond numbers, Equations (16) and (17) have to be solved numerically from $\phi =\frac{\pi }{2}-\theta \,$ to *φ* = 0 with the starting point for integration of ${x^{\prime} }_{I}=1$ and ${z^{\prime} }_{I}=0$. There is another unknown —${R^{\prime} }_{m0}$— in addition to *z*′ and *x*′ which has to be fixed before doing the integration. We use free energy analysis to find the appropriate ${R^{\prime} }_{m0}$, and consequently the meniscus shape and depth.

**Figure 3:**
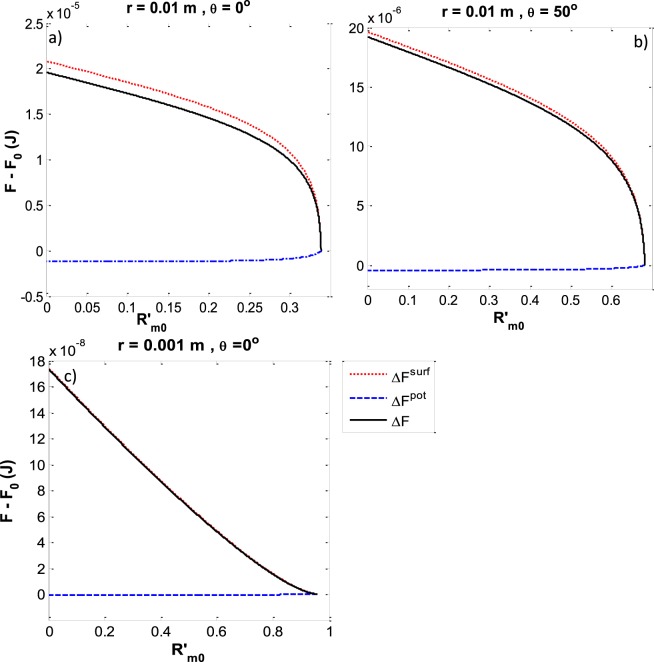
Free energy difference versus the reduced three-phase contact line curvature (${R^{\prime} }_{m0}$) for *r* = 0.01 m (**a**,**b**) and *r* = 0.001 m (**c**) and *θ* = 0° (**a** and **c**) and *θ* = 50° (**b**). The total energy difference (black lines) is composed of Δ*F*^*surf*^ (red dotted lines) and Δ*F*^*pot*^ (blue dashed lines). The total energy difference is a minimum when ${R^{\prime} }_{m0}$ is a maximum.

As is shown in Fig. 3 the free energy of the system is plotted for two different capillary sizes and contact angles. It shows that the potential energy is almost negligible especially for smaller capillary sizes and the surface energy almost governs the total energy difference. The trend of free energies shows that the largest acceptable $\,{R^{\prime} }_{m0}$ has the lowest free energy and consequently is more stable. As a result, we can generalized that for any size of capillary, the largest acceptable $\,{R^{\prime} }_{m0}$ is the correct meniscus curvature at the three-phase contact line.

**Figure 4:**
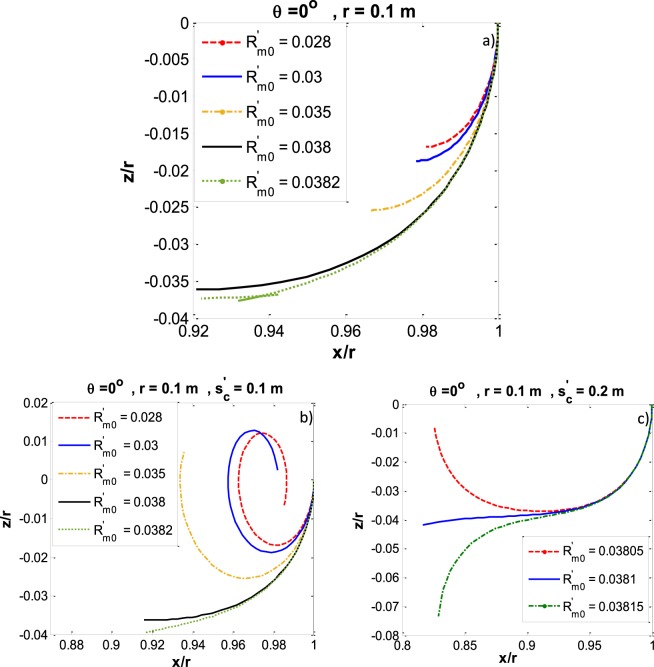
Capillary meniscus profile for zero contact angle and capillary radius *r* = 0.01 m for various $\,{{R}^{{\rm{^{\prime} }}}}_{m0}$ (**a**) *φ* as the integrating variable (**b**) *s* as the integrating variable and ${{s}^{{\rm{^{\prime} }}}}_{c}$ = 0.1 m c) s as the integrating variable and $\,{s{}^{{\rm{^{\prime} }}}}_{c}$ = 0.2 m.

In order to verify this generalization, we redid the calculation by considering “*s*” as the integrating variable. Figure 4 shows the capillary meniscus profile for a capillary with *r* = 0.1 m and zero contact angle for the liquid water – water vapour system. In Fig. 4(a), *φ* is the integrating variable and the meniscus profile is plotted for a series of $\,{R^{\prime} }_{m0}$. As is clear in Fig. 4(a) all $\,{R^{\prime} }_{m0}\le 0.038$ satisfy the conditions for equilibrium while $\,{R^{\prime} }_{m0}=0.0382$ is not acceptable. As a result, the correct reduced meniscus curvature at the three-phase contact line which is the largest acceptable $\,{R^{\prime} }_{m0}$ would be between 0.038 < $\,{R^{\prime} }_{m0}$ < 0.0382. Figure 4(b,c) shows the meniscus profile for the same system while *s* is the integrating variable. In the former case ${s^{\prime} }_{c}$ = 0.1 m and in the latter case ${s^{\prime} }_{c}=0.2$. It is shown that when 0.038 < $\,{R^{\prime} }_{m0}$ < 0.0382 the $\,{x^{\prime} }_{I}$ will tend to zero if ${s^{\prime} }_{c}$ increases properly.

**Figure 5:**
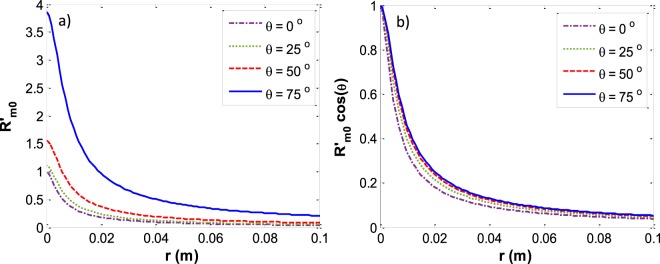
Reduced three phase contact line curvature (**a**) *R*′_*m0*_ (**b**) *R*′_*m0*_cos*θ* versus the capillary sizes at various contact angles.

In Fig. 5(a) the appropriate reduced curvature at the three-phase contact line is plotted for various capillary sizes and contact angles. The figure shows that $\,{R^{\prime} }_{m0}$ is decreasing with increasing capillary size and decreasing contact angle. In Fig. 5(b) the curvature at the three-phase contact line (*R*_*m*0_) is reduced by $\frac{r}{\cos \,\theta }$. When the entire meniscus has the shape of part of a sphere then the spherical curvature is ${R}_{mo,sph}=\frac{r}{cos\theta }$. It can be seen from Fig. 5(b) that all the menisci have an exactly spherical shape at small capillary sizes since ${R}_{mo}={R}_{mo,sph}$ which is equivalent to $\,{R}_{m0}^{{\rm{^{\prime} }}}\,\cos \,\theta$ going to one as capillary size becomes smaller and smaller.

**Figure 6:**
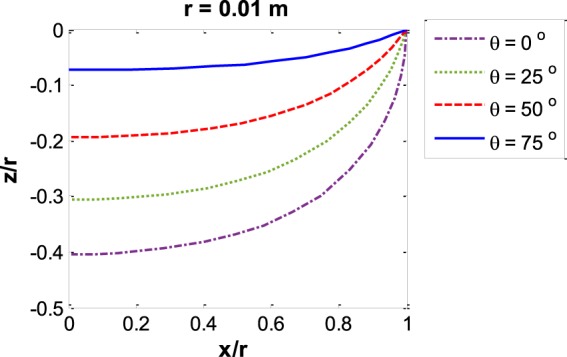
Capillary meniscus profile for various contact angles: *z*/*r* *vs*. *x*/*r* at constant capillary radius *r* = 0.01 m.

**Figure 7:**
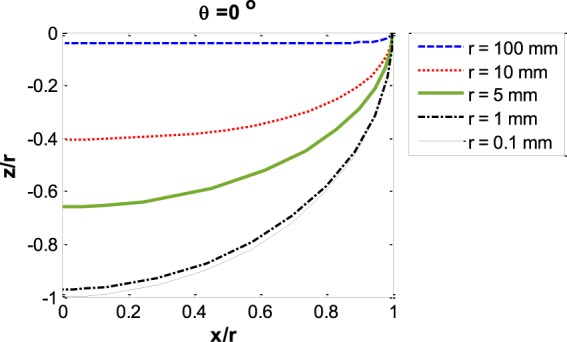
Capillary meniscus profile *z*/*r* *vs*. *x*/*r* for several capillary sizes at constant contact angle.

After finding the appropriate $\,{R^{\prime} }_{m0}$, the calculated meniscus profile can be plotted. Figure 6 shows *z*′ versus *x*′ for a capillary with radius of 1 cm for four different contact angles of 0°, 25°, 50° and 75°. It illustrates that at constant capillary size, the higher the contact angle, the smaller the reduced meniscus depth. Figure 7 shows *z*′ versus *x*′ for a zero contact angle meniscus at five different capillary radii of 0.1 mm, 1 mm, 5 mm, 10 mm and 100 mm. It can be seen that for small capillaries the meniscus forms part of a sphere and has the largest reduced meniscus depth while for large capillaries the inner majority of the meniscus is flat and the reduced meniscus depth is insignificant compared to capillary radius.

**Figure 8:**
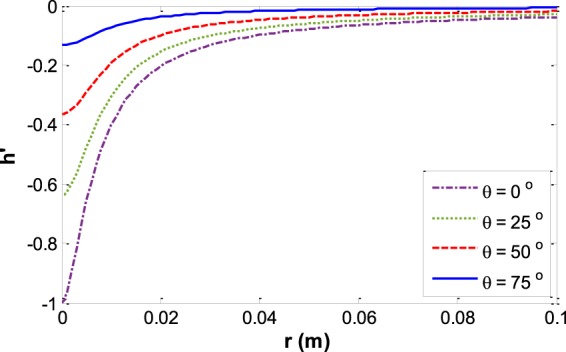
Reduced capillary depth *h′* versus capillary size *r* at various contact angles.

Figure 8 shows the highest reduced depth of capillary menisci at various contact angles and different capillary sizes. The highest depth of meniscus for each capillary happens at *φ* = 0, $h{}^{{\rm{^{\prime} }}}=z{}^{{\rm{^{\prime} }}}{|}_{\phi =0}$

In order to find the extent of deviation of the meniscus curvature from the spherical shape, we define a parameter *SR*. If the whole meniscus were part of sphere, as was explained in Fig. 5(b), ${R}_{mo,sph}=\frac{r}{\cos \,\theta }$. This is the case for small enough capillaries. In this case, the maximum depth of the capillary is:

38 $${h}_{sph}=\frac{r}{\cos \,\theta }(1-\,\sin \,\theta )$$

**Figure 9:**
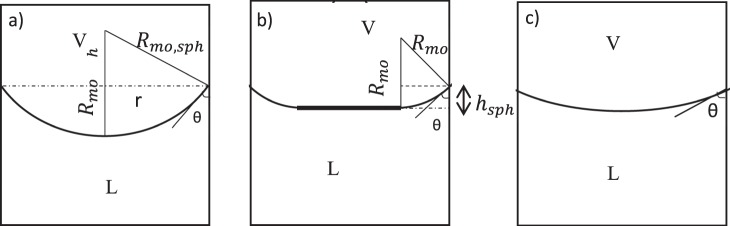
Three capillary menisci: (**a**) the whole meniscus is part of a sphere (**b**) part of the meniscus around the three-phase contact line forms a spherical shape (**c**) the meniscus is not part of a sphere at all.

However, if only a part of the meniscus around the three-phase contact line forms part of a sphere then the maximum depth of the capillary is: ${h}_{sph}={R}_{mo}(1-\,\sin \,\theta )$. These cases are shown in Fig. 9. As a result, the parameter $SR=|\frac{{h^{\prime} }_{sph}}{h^{\prime} }|=|\frac{{R^{\prime} }_{mo}(1-\,\sin \,\theta )}{h^{\prime} }|$ is an illustration of the deviation of the three phase contact line curvature from the spherical shape.

**Figure 10:**
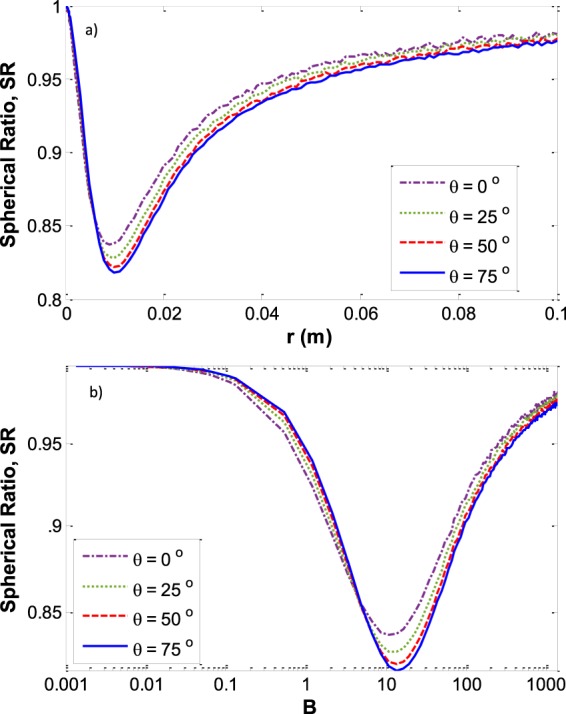
The deviation of three-phase contact line curvature from a spherical shape. (**a**) *SR* versus *r* at various contact angles (**b**) *SR* versus *B* on a logarithmical scale at various contact angles for the liquid water – vapor water system.

Figure 10 shows the SR versus the Bond number on a logarithmical scale (Fig. 10b) and versus capillary size (Fig. 10a) at various contact angles. It shows that for all contact angles the highest deviation from spherical curvature at the three-phase contact line (the higher SR) happens for a capillary size of around 1 cm or more generally at Bond numbers of around 13.

**Figure 11:**
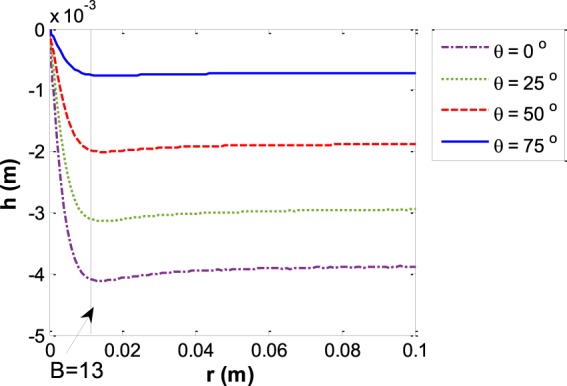
Meniscus depth *vs*. the capillary size for various contact angles for the liquid water – water vapor system.

As was shown previously in Fig. 5b, for small capillaries the whole menisci form part of a sphere. Therefore, the *SR* is equal to 1 for small capillary sizes. By increasing the size of the capillary the three phase contact line curvature deviates more and more from the spherical shape and the *SR* becomes smaller than one. At some specific capillary size the three-phase contact line curvature has the maximum deviation from spherical and after that the deviation decreases more and more until the *SR* again approaches approximately to one. For large capillaries, although there is a large flat portion of the interface around the center of the meniscus, there exists approximately spherical curvature at the three phase contact line. Figure 11 shows the capillary depth versus the capillary size at various contact angles.

**Figure 12:**
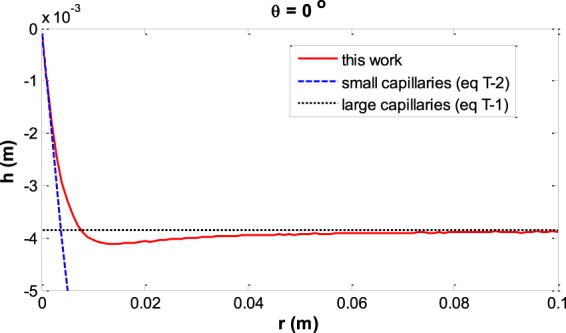
Validation of the results of this work in the limit of small and large capillaries based on the equations in Tables 1 and 2.

In order to validate the results, for large enough capillaries we can estimate our result by the meniscus depth facing a vertical plate which is:

39 $${h}_{ver-plate}=\sqrt{2\frac{{\gamma }^{LV}}{\Delta \rho g}(1-\,\sin \,\theta )}\,$$

and was verified by experiment^32^. For small capillaries, as we expect the depth of the meniscus is equal to the capillary radius for zero contact angle and in general it is equivalent to Equation (38). The numerical values for these validations are shown in Tables 1 and 2 and Fig. 12 shows this validation for the liquid water – water vapor system with contact angle of zero.

**Table 1:** Validation of numerical calculation for large enough capillaries.

Contact angle *θ*	Meniscus depth facing a vertical plate^32^: Eq. (39)	Meniscus depth in large capillaries, *i*.*e*., *r* = 0.1 m
0°	*h* = 3.86 × 10^−3^ m	*h* = 3.88 × 10^−3^ m
25°	*h* = 2.93 × 10^−3^ m	*h* = 2.95 × 10^−3^ m
50°	*h* = 1.86 × 10^−3^ m	*h* = 1.88 × 10^−3^ m
75°	*h* = 0.711 × 10^−3^ m	*h* = 0.719 × 10^−3^ m

**Table 2:** Validation of numerical calculations for small enough capillaries.

Contact angle *θ*	Meniscus depth assuming spherical shape *r = *1 × 10^−4^ m: Eq. (38)	Meniscus depth in small capillaries, *i*.*e*., *r = *1 × 10^−4^ m
0 °	*h* = 1 × 10^−4^ m	*h* = 9.9968 × 10^−5^ m
25°	*h* = 6.3707 × 10^−5^ m	*h* = 6.3697 × 10^−5^ m
50°	*h* = 3.6397 × 10^−5^ m	*h* = 3.6393 × 10^−5^ m
75°	*h* = 1.3165 × 10^−5^ m	*h* = 1.3164 × 10^−5^ m

### Water – soybean oil system

**Figure 13:**
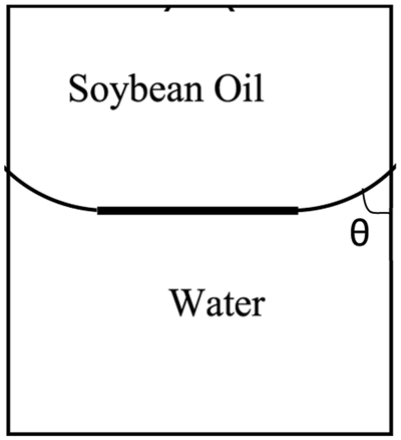
Liquid water – soybean oil meniscus.

**Figure 14:**
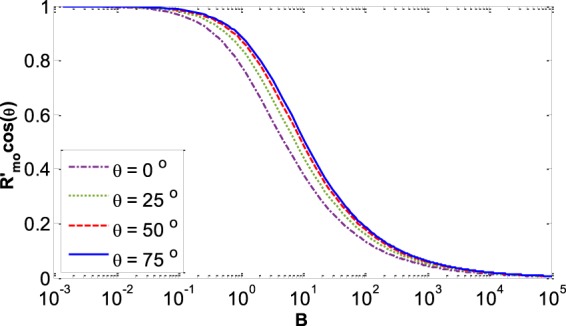
The general form of *R′*_*mo*_*cosθ* for different contact angles for both systems of liquid water–water vapor and liquid water–soybean oil menisci.

**Figure 15:**
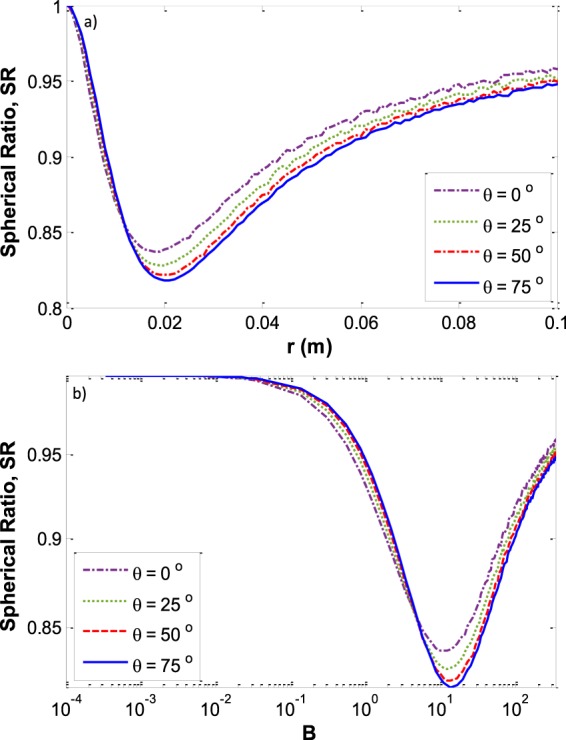
The deviation of the three-phase contact line curvature from spherical. (**a**) *SR* *vs*. *r* at various contact angles; (**b**) *SR* *vs*. *B* on a logarithmical scale at various contact angles for the water – soybean oil system.

Here we make calculations for a meniscus with very different physical properties, a liquid–liquid meniscus rather than a liquid–vapor meniscus. Recently, the meniscus between oil and water has new important applications such as in slippery lubricant impregnated surfaces^33,34^ or microfluidic applications^35^ where usually vegetable oils such as soybean oil, silicon oil, or peanut oil are used as a lubricant. We found the meniscus shape for water and soybean oil in a capillary with radius *r* and contact angle of the water–soybean oil meniscus with the capillary wall of *θ* measured through the water (Fig. 13). The physical properties of this system^36^ are: *T* = 25 °C, ${\rho }^{W}=997.04\frac{{\rm{kg}}}{{{\rm{m}}}^{3}}$, ${\rho }^{SO}=917.47\frac{{\rm{kg}}}{{{\rm{m}}}^{3}}$, ${\gamma }^{LV}=0.0228\frac{{\rm{J}}}{{{\rm{m}}}^{2}}$. where *ρ*^*W*^ is the density of the liquid water phase and *ρ*^*SO*^ is the soybean oil density. It is found that for both systems of liquid water – water vapor and water – soybean oil the plots of *R′*_*mo*_*cosθ* versus the Bond number for different contact angles follow the general trend which is shown in Fig. 14. Figure 15 for the water – soybean oil meniscus is similar to Fig. 10 for the liquid water – water vapor meniscus. The figure illustrates that for either of these systems the maximum deviation from spherical curvature at the three-phase contact line happens around a Bond number of 13, while because of the different system physical properties the maximum deviation for the water – soybean oil system happens at a capillary radius of 2 cm. Since the Bond number includes all the physical properties involved in determining meniscus shape—including the capillary size, surface tensions and density differences between the phases—it is the most comprehensive variable to be investigated in these types of problems.

**Figure 16:**
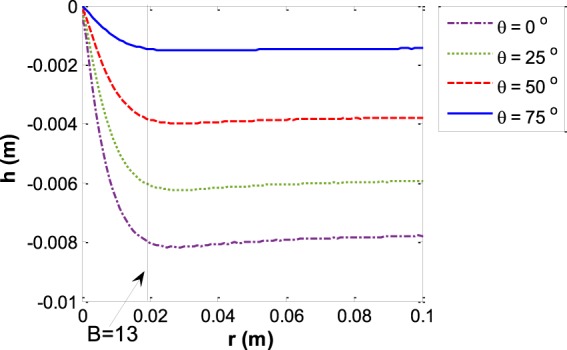
Meniscus depth *vs*. the capillary radius for various contact angles for the water – soybean oil system.

**Figure 17:**
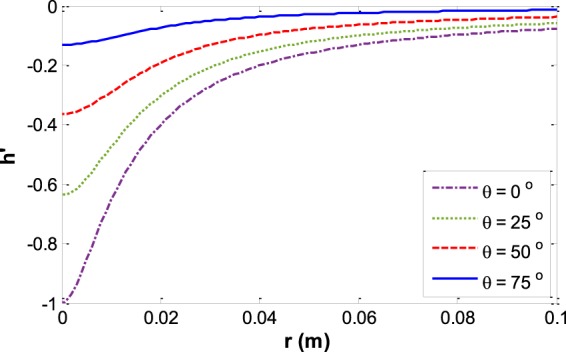
Reduced capillary depth *h*′ versus capillary size *r* at various contact angles for the water – soybean oil system.

Figure 16 shows the depth of water menisci with an oil phase for various contact angles and different capillary sizes and the trends are similar to those for the water – water vapor system. Figure 17 indicates the reduced capillary depth *vs*. the capillary size for several contact angles for water – soybean oil menisci. By increasing the capillary size the reduced capillary depth increases while the capillary depth decreases.

### Potential application: Measurement of surface tension by the capillary rise method (low surface tension or large capillaries)

One of the oldest methods to measure surface tension is the capillary rise method. In cases for which *Bo* < 0.01 this method is easily accessible. However, usually this criteria is hard to achieve and for larger Bond numbers, Sugden^37^ and later Lane^38^—based on the Bashforth and Adams work—prepared a table/graph of *r/a* versus *r/b* where *r*, *a* and *b* are the capillary radius, capillary length $(\sqrt{\frac{2{\gamma }_{LV}}{{\rm{\Delta }}\rho g}})$ and curvature of the meniscus at the apex. However, their work is restricted to a contact angle of zero and did not cover the ranges of system parameters for which a portion of the interface is flattened. Importantly this restriction means that the capillary rise method could not be used to measure low surface tension values—more expensive specialized equipment such as the spinning drop tensiometer is typically used, which introduces its own difficulties^39^.

**Figure 18:**
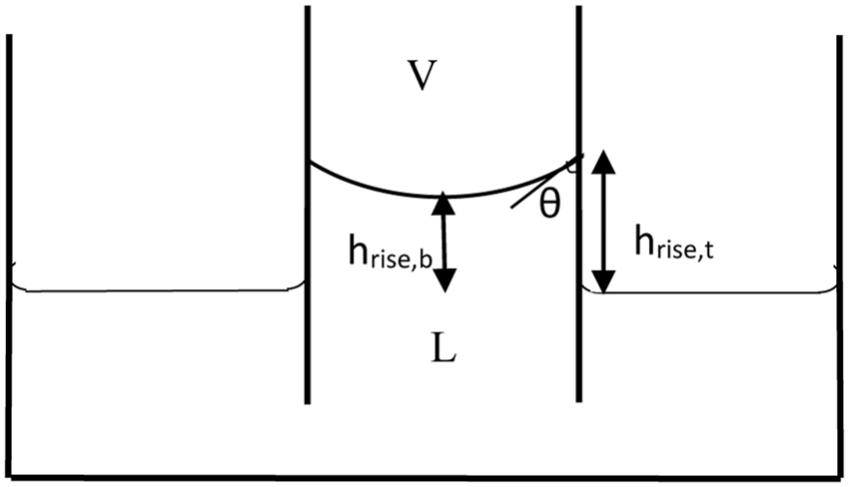
Capillary rise schematic.

As shown in Fig. 18, the capillary rise can be determined from the three-phase contact line (*h*_*rise*,*t*_) or from the meniscus apex (*h*_*rise*,*b*_). The latter is usually measured due to the use of the Bashforth and Adams table which tabulated the meniscus curvature at the apex. Although working with the former capillary rise is not usual, it can be used in measuring the surface tension in small capillaries and even in larger capillaries in which the meniscus has a flattened center where *h*_*rise*,*b*_ = 0, which will be the case for low surface tension values. These two capillary rises are related by following equation:

40 $${h}_{rise,b}b={h}_{rise,t}{R}_{mo}=\frac{2{\gamma }^{LV}}{{\rm{\Delta }}\rho g}$$

**Figure 19:**
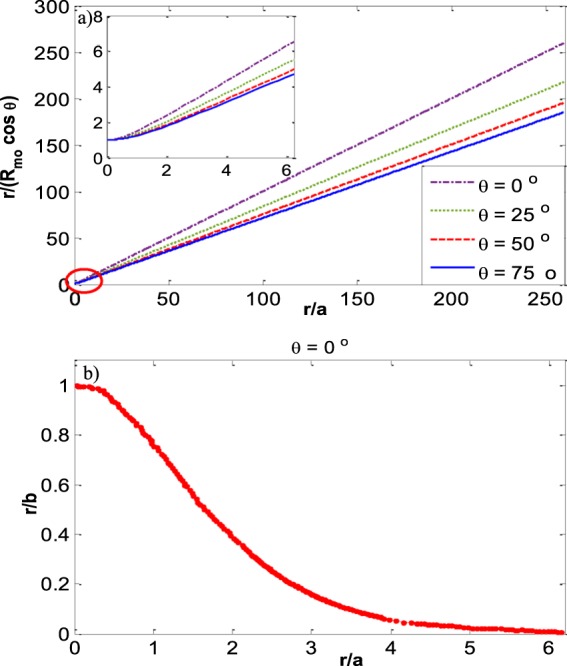
(**a**) Inverse of meniscus curvature at the three-phase contact line *r/(R*_*mo*_*cosθ)* versus *r/a* for different contact angles. The red oval region is expanded in the inset (**b**) inverse of meniscus curvature at the apex versus *r/a* at zero contact angle (reproduced from the work of Sugden^37^ & Lane^38^).

In fact, in the case of a flat interface we do not have any capillary rise at the apex (*h*_*rise*,*b*_ = 0) but there is still a capillary rise at the three-phase contact line (*h*_*rise*,*t*_ ≠0). Our approach provides a means to measure the surface tension even in cases that there is a big container, or a low surface tension. Figure 19 shows the inverse of meniscus curvature versus *r/a* for different contact angles. Figure 19(b) is reproduced from the work of Sugden and Lane’s and the meniscus curvature is at the apex (*b*) while in Fig. 19(a) the meniscus curvature is at the three-phase contact line. It should be noted that since all the menisci with various contact angles have a spherical shape at small sizes—to have them all start at the same position we have multiplied *R*_*mo*_ with *cosθ*.

**Figure 20:**
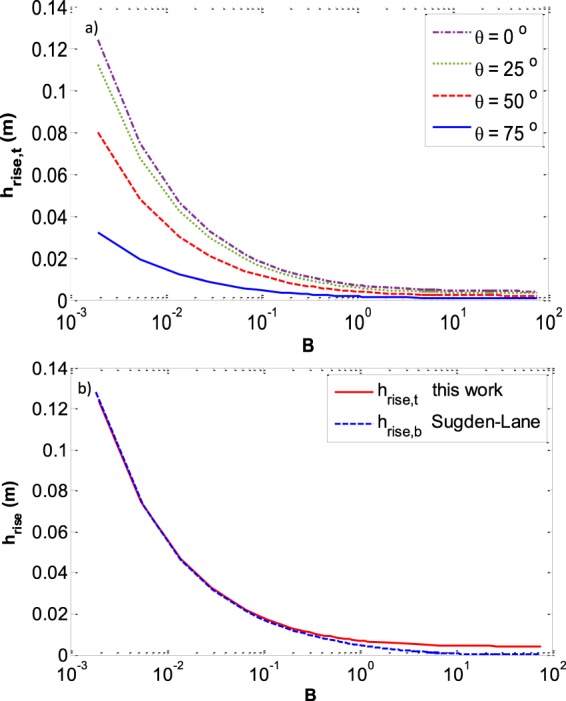
(**a**) Capillary rise at the three-phase contact line for various contact angles (**b**) Capillary rise at the three-phase contact line and at the meniscus apex for *θ* = 0.

Figure 20 shows the capillary rise versus the Bond number. In Fig. 20(a), the capillary rise at the triple contact line is plotted for various contact angles and in Fig. 20(b) both *h*_*rise*,*t*_ and *h*_*rise*,*b*_ are plotted for a contact angle of zero. As we expect, for smaller capillaries *h*_*rise*,*b*_ and h_rise,t_ coincide since the gravity effect is negligible and the pressure difference throughout the whole interface is the same. However, by increasing the size of the capillary, or decreasing the surface tension, both of which increase the Bond number, the deviation between *h*_*rise*,*b*_ and *h*_*rise*,*t*_ becomes important and in the case of a flatted central part of the interface, although *h*_*rise*,*b*_ is equal to zero and cannot be used for surface tension measurement, *h*_*rise*,*t*_ has a determined value that can be used to measure interfacial tension.

**Figure 21:**
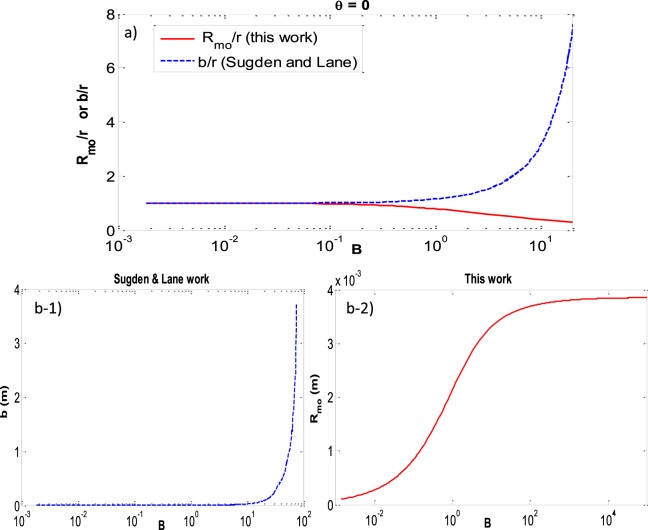
(**a**) Reduced curvature at the apex (*b/r*) versus Bond number (blue dashed line); reduced curvature at the three-phase contact line (*R*_*mo*_*/r*) versus Bond number (red solid line) **b -1**) curvature at the apex (*b*) versus Bond number (blue dashed line); **b -2**) curvature at the three-phase contact line (*R*_*mo*_) versus Bond number (red solid line).

Figure 21(a) shows the reduced curvature at the apex (based on the work of Sugden and Lane) and the reduced curvature at the three-phase contact line. By increasing the Bond number the apex curvature increases more and more to reach the flat interface—with radius of curvature going to infinity—(Fig. 21-b-1) while the curvature at the three-phase contact line increases at such a slower rate (Fig. 21-b-2) that it leads to the decrease in its reduced curvature.

## Conclusion

In this study the capillary meniscus depth is evaluated for various capillary radii and different contact angles for two systems with different properties: the water liquid – water vapor system and the water – soybean oil system. In order to integrate the Young–Laplace equation numerically, we have changed the typical starting point for the integration from the apex of the interface as is used in typical interface shape calculations to be instead at the three-phase contact line, since the apex point cannot be used as the starting point for integration to predict the meniscus shape for systems with larger Bond numbers where the meniscus has some central portion flattened. The curvature at this new integration starting point—at the three-phase contact line—is evaluated by free energy calculation. It was found that the curvature at the three-phase contact line would be the highest acceptable curvature and this value correspond to the smallest system free energy. The same result is obtained if the curvature length (*s*) is the integrating variable instead of turning angle (*φ*) as the integrating variable. The deviation of curvature at the three-phase contact line from that of a spherical shape is examined and it was found that the highest deviation which is about *SR* = 0.83 happens at a Bond number of approximately 13 for both systems studied. As a result, if the curvature at the three phase contact line is unknown—which is the pre-requirement to solve the Young–Laplace equation—it can be estimated with spherical shape with the deviation reported here. Finally, using the numerical methods presented here to calculate the shapes of capillary menisci in the two systems for various values of capillary radius and contact angle, insight is gained into the dependence of meniscus depths on the various variables. One of the potential applications of this approach is to measure the capillary rise at the three-phase contact line to find the surface tension in high Bond number system such as systems with large capillaries or low surface/interfacial tensions.
